# Building perception block by block: a response to Fekete *et al.*

**DOI:** 10.1093/nc/niy012

**Published:** 2019-01-28

**Authors:** Adrien Doerig, Frank Scharnowski, Michael H Herzog

**Affiliations:** 1 Laboratory of Psychophysics, Brain Mind Institute, École Polytechnique Fédérale de Lausanne (EPFL), Switzerland; 2 Department of Psychiatry, Psychotherapy and Psychosomatics, Psychiatric Hospital, University of Zürich, Lenggstrasse 31, Zürich, Switzerland; 3 Neuroscience Center Zürich, University of Zürich and Swiss Federal Institute of Technology, Winterthurerstr. 190, Zürich, Switzerland; 4 Zürich Center for Integrative Human Physiology (ZIHP), University of Zürich, Winterthurerstr. 190, Zürich, Switzerland; 5 Department of Basic Psychological Research and Research Methods, Faculty of Psychology, University of Vienna, Liebiggasse 5, Vienna, Austria

**Keywords:** consciousness, discrete perception

## Abstract

Is consciousness a continuous stream, or do percepts occur only at certain moments of time? This age-old question is still under debate. Both positions face difficult problems, which we proposed to overcome with a 2-stage model, where unconscious processing continuously integrates information before a discrete, conscious percept occurs. Recently, Fekete *et al.* criticized our model. Here, we show that, contrary to their proposal, simple sliding windows cannot explain apparent motion and related phenomena within a continuous framework, and that their supervenience argument only holds true for qualia realists, a philosophical position we do not adopt.

Intuitively, consciousness is a continuous stream of percepts. We see a diver jumping from a cliff into the ocean and have the feeling we perceive their motion trajectory at each single moment of time. However, continuous perception theories face serious problems known since ancient times. For example, a disk is presented at two locations separated by a blank period (Fig. 1). We do not perceive *two* small disks presented at *two* locations, but a *single* disk moving between the locations even though there is no motion at all in the stimulus (apparent motion; Fig. 1). Obviously, we can only perceive the motion after the second disk is presented and, hence, perception cannot be immediate. Other examples demonstrating that the percept cannot occur immediately include feature fusion, the flash-lag illusion, the continuous wagon-wheel illusion, etc.

**Figure 1.**
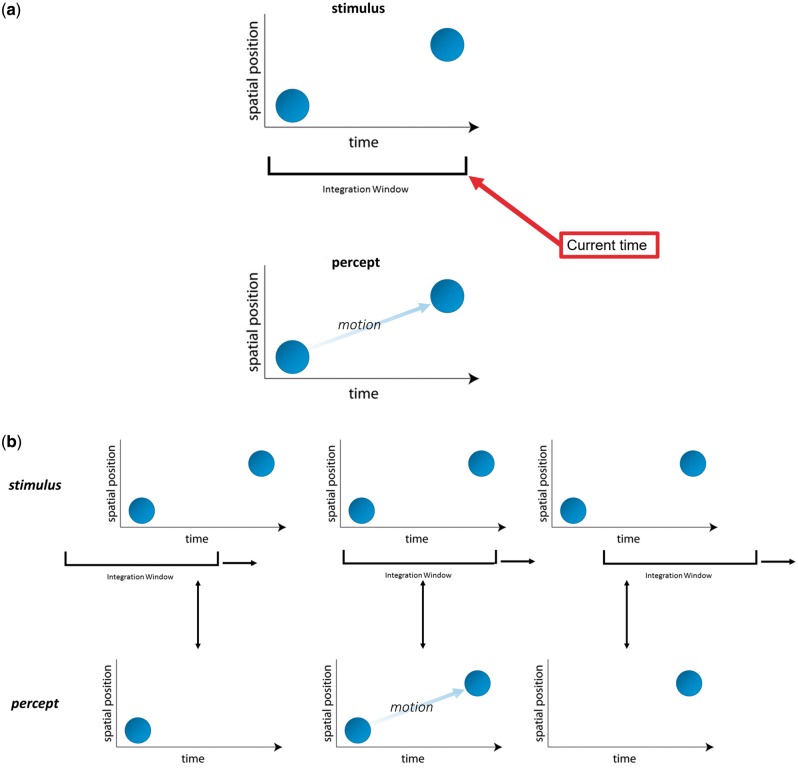
(**a**) Apparent motion: In apparent motion, two disks are flashed at different positions separated by a blank period (top). We do not perceive two distinct disks but a single moving disk (bottom). Hence, both disks must be integrated before a percept is created. Hence, an integration period is needed spanning at least the duration of the two disks. (**b**) Sliding windows can explain why we perceive discrete events as continuous, but not the discreteness of perception. In a sliding window account of apparent motion, before the stimulus is presented, a blank screen would be perceived (not shown). Then, the window reaches the first disk, so we would perceive a static disk (left). Next, both disks fall into the integration window, thus activating motion detectors, and we would perceive continuous apparent motion (centre). Finally, only the second disk is in the window and we would perceive a second static disk (right). However, we only perceive one moving disk. Hence, sliding windows can explain why apparent motion appears to be continuous (i.e. discrete events are perceived as continuous) but *not* why we do not perceive static disks before and after the motion (i.e. perception is discrete).

To accommodate these findings, discrete theories propose that percepts occur only at certain moments of time. For example, snapshot theories propose that we sample information from the environment like a surveillance camera, taking pictures periodically. However, these positions also face severe problems (see Herzog *et al.* 2016). In particular, no experiment has ever shown evidence for a *unique* and paradigm-independent sampling rate. In addition, any information between snapshots would be lost.

To overcome these problems, we proposed a 2-stage theory (Herzog *et al.* 2016) where continuous, unconscious processing with high temporal resolution integrates information for several hundred milliseconds (Stage 1), which is then rendered conscious as a coherent percept at a discrete moment in time (Stage 2). Importantly, temporal features, such as motion, are not consciously perceived *while* they occur. They are not even perceived over an extended period of time, but are encoded as any other feature, such as colour or shape, by a *static* label. For example, motion is not represented by a signal that moves in time but by the output of a motion detector.

Fekete *et al.* (2018) criticized our model based on two main arguments.

1. Sliding windows: They argue that phenomena such as apparent motion can be explained within a continuous framework by sliding windows. We will explain why this argument fails.
2. Perceptual change and neural change: They claim that perceptual change must be mirrored by neural change: ‘admitting that there is perceptual change (e.g. in the location of the object) not mirrored in neuronal change [_**…**_] would amount to violation of supervenience—the notion that consciousness is determined by physical processes [_**…**_]’. We will show that either there is a misunderstanding about supervenience in their argument or they subscribe to a realist position about qualia.

## Sliding windows

There is agreement that ‘instantaneous’ continuous theories, in which sensory evidence is immediately translated into a conscious percept cannot be true because phenomena such as apparent motion require integration over extended periods of time. Fekete *et al.* proposed that continuous theories can explain such phenomena by sliding windows (Fig. 1). For example, a window, starting integration with the presentation of the first disk and terminating with the second one, might explain apparent motion. Conscious perception just occurs after the presentation of the second disk—it is delayed but continuous (Fig. 1a). However, the example fails for a very simple reason. Integration does *not* start with the presentation of the first disk and terminates with the second one. It is continuous! Let us consider—step by step—what would happen in the sliding window account (Fig. 1). First, the window comprises only an empty screen and, hence, only an empty screen would be perceived. When we move the window further, it comprises empty screen moments and the first disk. At this stage, we would perceive the first disk—and only it. When we move the window further, both disks are now present, and we would perceive motion. Finally, we would perceive only the second disk when the first disk is outside the sliding window. However, this is not what we actually perceive. We see only one moving disk and never single static disks. Hence, the idea of a rigid sliding window is not tenable.

As mentioned, a sliding window can explain why we perceive motion *when the window contains both disks*. For example, a classical motion detector fires only when it is stimulated by two distinct consecutive events (for certain spatial positions and delays, thus, creating a direction and speed sensitive neuron; Adelson and Bergen 1991; Jancke *et al.* 2004; Lu and Sperling 1995; Watson and Ahumada 1985). Such a motion detector is equivalent to an integration window and explains why we perceive two discrete events as continuous, as is the case in apparent motion. Hence, it explains why we perceive continuity instead of discrete events. However, it does not explain the discreteness of perception. Namely, it cannot explain why we only perceive a *single* moving disk and *not* two additional static disks in windows preceding and following the window containing both disks—even though the visual system clearly can detect both the first and second disk when they are presented alone (e.g. using a ‘static disk’ detector). The very same argument applies to feature fusion and other phenomena where two discrete events are perceived as a single continuous event.

## Perceptual change and neural change

We completely agree with Fekete *et al.* that different percepts must come with different brain states. It cannot be that the very same brain state gives rise to different percepts. This is a pre-requisite for studying consciousness neuroscientifically. However, this does not imply that *temporal* changes in perception, such as motion, are mirrored by *temporal* changes in the brain. Motion in the external world does not need to be represented by brain *dynamics*—it can simply be coded statically by the output of a motion detector. Different kinds of motion (speed, direction, etc.) are encoded by different motion detectors. Likewise, a 40 ms stimulus may not be perceived over a duration of 40 ms but instead might be encoded by the output of a duration detector, i.e. a ‘static’ number indicating a 40 ms duration. And a duration of 50 ms might be encoded by another detector. According to our theory, different percepts correspond to different brain states. Thus, there is no supervenience problem. It seems that Fekete *et al.* postulate that each physical moment of motion needs to be represented by a different brain state, potentially because they subscribe to a realist position about qualia (see Qualia and philosophical assumptions section of this contribution). Accordingly, when we perceive a ball moving continuously from A to B, there must be a *different* brain state for each intermediate position of the ball. In our approach, this is not the case—a *single* brain state encodes the entire motion percept.

## Related arguments

Subsequent arguments against our model rest on this misunderstanding of the supervenience argument, or on strong qualia realism. For example, Fekete *et al.* argue that, if perception were discrete, updates would need to occur roughly every 33 ms (i.e. at 30 Hz, but the precise value is not crucial) because neural change needs to match perceptual change. They further argue that this is impossible because of the slow neural dynamics of the primary visual cortex. However, this argument does not apply to our theory for the reasons described above.

## Qualia and philosophical assumptions

The question about the time course of perception directly relates to the question of qualia. As mentioned, motion *detection* does not need to be coded by a dynamically changing representation. What about motion *experiences*? In our model, the experience of motion does not extend in time, it only *seems* to. In this respect, we are close to the illusionist position (Dennett 2016; Frankish 2016), which proposes that qualia do not exist as real distinct entities—they only *seem* to. Similarly, the meta-problem research program (Chalmers 2018) aims to explain why we think there is a hard problem of consciousness, i.e. why it *seems* that qualia exist as real distinct entities.

Our approach is at odds with a realist interpretation of qualia, which Fekete *et al.* seem to adopt. Given such incompatible philosophical underpinnings, our proposals are naturally very different. Thus, our dispute gets to the heart of the heated debate on how to link neural processes to conscious percepts. Because the sliding window argument fails to account for phenomena in which two discrete events are perceived as a single continuous event (and for other independent reasons, e.g. Sergent 2018), we suggest that discrete models should be favoured. Whatever the final answer is, we believe that questions about the time course of conscious perception, a highly under-investigated research area, are a fundamental stepping-stone to understand perception and consciousness, and we thank Fekete *et al.* for their stimulating contribution to this crucial debate.
